# Factors Affecting the Consumption of Traditional Food in Tourism—Perceptions of the Management Sector of Catering Facilities

**DOI:** 10.3390/foods12122338

**Published:** 2023-06-10

**Authors:** Bojana Kalenjuk Pivarski, Dragana Tekić, Stefan Šmugović, Maja Banjac, Aleksandra Novaković, Beba Mutavdžić, Velibor Ivanović, Dragan Tešanović, Bojan Đerčan, Predrag Ikonić, Marica Petrović, Dragana Ilić Udovičić, Aleksandra Vasić Popović, Aleksandar Marić

**Affiliations:** 1 Department of Geography, Tourism and Hotel Management, Faculty of Sciences, University of Novi Sad, 21000 Novi Sad, Serbia; bojana.kalenjuk@dgt.uns.ac.rs (B.K.P.); stefan.smugovic@dgt.uns.ac.rs (S.Š.); maja.banjac@dgt.uns.ac.rs (M.B.); veliborivan98@gmail.com (V.I.); dragan.tesanovic@dgt.uns.ac.rs (D.T.); bojan.djercan@dgt.uns.ac.rs (B.Đ.); 2 Faculty of Economics, University of East Sarajevo, 71420 Pale, Bosnia and Herzegovina; 3 Department of Agricultural Economics and Rural Sociology, Faculty of Agriculture, University of Novi Sad, 21000 Novi Sad, Serbia; dragana.tekic@polj.uns.ac.rs (D.T.); marica.petrovic@polj.uns.ac.rs (M.P.); 4 Faculty of Education, University of East Sarajevo, 76300 Bijeljina, Bosnia and Herzegovina; aleksandra.novakovic@pfb.ues.rs.ba; 5 Institute of Food Technology, University of Novi Sad, 21000 Novi Sad, Serbia; predrag.ikonic@fins.uns.ac.rs (P.I.); aleksandar.maric@fins.uns.ac.rs (A.M.); 6 Academy of Professional Studies Šabac, Department of Medical and Business-Technological Studies, 15000 Šabac, Serbia; dilicudovicic@vmpts.edu.rs (D.I.U.); aleksandra.vasic@vmpts.edu.rs (A.V.P.)

**Keywords:** food perception, catering, traditional food, tourism, Vojvodina

## Abstract

The topic of this study is the factors that influence the consumption of traditional food products (TFPs) in tourism, as seen from the perspective of management-sector employees in food and beverage catering facilities. The paper aims to analyse the economic, environmental, social, and touristic factors that highly influence the consumption patterns of catering facilities which are significant providers of traditional gastronomic experiences in tourism, by using the specially designed TFPct scale. The study was conducted on a sample of 300 catering facilities in AP Vojvodina (the Republic of Serbia). An explanatory factor analysis was used to confirm the key factors that influence the consumption of traditional products used to prepare the meals that are a part of what catering facilities offer. Subsequently, a binary logistics regression model was used to establish which of the indicated factors has a statistically significant effect on the management’s decision to purchase these products for their catering facility. The study showed that the TFPct scale is appropriate for this type of research, and that economic factors are key factors in the consumption of traditional products. Moreover, compared with other types of catering facilities, interest in the consumption of these products is clearly expressed by a la carte restaurants.

## 1. Introduction

Traditional food products (TFPs) are an important part of the culture, identity, and heritage of every nation [1,2,3,4]. They are products which are prepared from specific ingredients, using special meal preparation methods passed down from generation to generation [5]. As such, TFPs are the complete opposite of mass-produced food, because in their case the focus is on the importance of the product as a part of cultural heritage [6]. TFPs are constantly being revived by increased supply and demand from consumers and different commercial entities that are aware of their significance [1]. In scientific research, there are different approaches to studying TFPs [7,8]. Their traditionalism is linked with their production method or with labels that point out their idiosyncrasy [9]. As a result, consumers create a particular image and associate these products with terms such as “traditional cuisine” and “traditional recipes” [5]. Due to their uniqueness and specificity, TFPs are highly desirable elements of the catering offerings in tourism [1,2,3,4]. By offering TFPs, catering facilities are helping to revitalize the countryside and economy, thus stimulating the development of tourism and the promotion of gastronomical identity. This in turn directly influences the economic and social development of many regions and areas [10,11,12,13]. 

It is important to point out that the consumption of TFPs is directly conditioned by the supply and demand chain in catering facilities, which in turn influences the management sector of catering facilities to acknowledge the importance of these products [14]. This is one of the reasons why employees in this sector were surveyed on this topic.

This paper analyses the factors (environmental, cultural, touristic, and economic) that affect TFP consumption in tourism, as seen from the perspective of management-sector employees in various food and beverage catering facilities. It focused on products labelled TASQ (Traditional and Standard Quality), which are typical for the studied area (AP Vojvodina, the Republic of Serbia). These products come with a certificate that proves their sensory or nutritional quality. These documents include additional indicators of quality, such as the label “organic product”, various geographical labels, autochthonous/wild sort/type/species labels, labels proving traditional production means and certified HACCP, and labels indicating that the production took place on family farms [15].

The paper aimed to analyse the factors that influence the consumption of TFPs in catering facilities in AP Vojvodina (the Republic of Serbia) based on similar studies on the given topic [3,4,16,17].

## 2. Literature Review

### 2.1. Traditional Food

TFPs are made by processing and producing different ingredients originating from a certain area [3,4]. They are famous for their production recipe and the origin of the raw materials which are used in their preparation, or in other words, their entire production process. Their presence on the market needs to date back at least 50 years, and of course they have to be a part of the gastronomic heritage [18]. Due to the specific nature of their production process, TFPs are famous for their distinctive sensory and/or nutritional quality [4,19,20]. The quality of food and its production process are some of the most important food-related characteristics nowadays. This is precisely where TFPs stand out in relation to standardized mass-produced food. The mere notion of food being processed in smaller amounts raises awareness about using fewer preservatives and other substances which influence quality sustainability. This is why they can be found in smaller amounts, only in certain seasons, as well as in certain areas [21]. In this way, food production, or mass food production to be precise, has shifted focus to the revaluation of the gastronomic tradition and the preservation of traditional culinary values [22] that are important for tourism.

TFP production contributes to the improvement of the food offer in developing countries. These products, either of plant or animal origin, produced on local farms and fields, help create a specific ethical bond regarding the creation of healthy and natural dietary habits [23].

### 2.2. The Importance of the Perception of Food and the Factors Which Impact Consumption 

The manner in which employees in catering facilities perceive authentic and local food products has a significant impact on their business behaviour models and on creating the gastronomic offer. Robinson et al. [24] point out that in developing countries hospitality workers are seen as actors who contribute to the strengthening and development of tourism; however, this is not always the case in practice. In more developed areas, they represent a significant component of the tourist infrastructure which shapes the tourist product [24]. 

TFPs, as a reflection of the geographical heritage of a certain region, represent an economic and cultural phenomenon which relies on the production and consumption of food, and forges a connection between consumers and producers, protecting all the subjects involved in the process [25]. It is precisely for this reason that the paper analyses ecological, socio-cultural, economic, and touristic factors as the topic of study, along with the significant elements in the perception of hospitality workers who procure them and offer them to their consumers. 

#### 2.2.1. Environmental Factors

The production of TFPs in a certain area affects the preservation of the ecosystem, which raises the awareness of local residents about the need to protect the environment. Unlike conventional products, TFPs not only have significantly fewer negative impacts on the region, such as noise, environmental pollution, and the degradation of natural resources, but also reconstruct natural heritage, which in this case remains intact [26].

The impact of food and nutrition on human health and the environment is one of the key topics discussed in political and social circles. It is frequently discussed among researchers and conservationists alike, because food production leads to a significant increase in global greenhouse gas emissions. Food production processes are also the main consumer and polluter of water [27,28]. That is why it is often pointed out that food production and food consumption should originate from the same locality [29].

As a result, marketing strategies often focus on the debate about reducing pollution which occurs if food is produced and consumed in the same localities. In the case of TFPs, because they are produced by means of old methods or from old plant and wildlife species, they are often not even suited to large-scale distribution [30]. Here we ought to mention the use of PVC packaging, which is significantly less frequent in the case of TFPs than it is in the case of mass-produced products [31]. From an environmental point of view, the largest percentage of plastic used for food packaging ends up in wastewater shortly after purchase. This is particularly true of disposable packaging, because it is not disposed of properly [32,33].

#### 2.2.2. Socio-Cultural Factors

Socio-cultural factors in TFP use include the preservation of tradition, cultural identity, customs, and folklore, as well as local gastronomy, i.e., gastronomic heritage [34,35]. Activating the production and consumption of these products helps to activate young people, preventing the migration of the local population from rural areas, which helps the local community improve their quality of life, i.e., improve the living standards [7,36,37,38]. 

Moscatelli et al. [6] define social sustainability as the ability to establish continued quality of life and human well-being, while taking into account the tradition that gives it an original, unique identity. The term “tradition” in relation to food and gastronomy is closely associated with elements such as time and knowledge, that is, culture, which includes the dimension of place or locality of the food. Here, the concept of TFPs includes terms such as typical food, specialties, and heritage food [12,39,40].

#### 2.2.3. Economic Factors

Studies have confirmed that the use of TFPs increases the demand for regional restaurants, which has a considerable economic impact [41,42]. Therefore, the production of TFPs is an important factor in economic development which impacts the diversification of rural areas [43], promotes sustainable agriculture and craftsmanship, decreases regional economic gaps, and strengthens local economies [44,45]. However, it also leads to a reduction in depopulation [43,46]. The market battle between global brands and TFPs is constant [47]. Florek and Gazda [22] state that only global brands can bring an economic effect. Nevertheless, it is worth pointing out that TFPs have an impact on the creation of a unique value and play an important role in creating a brand for their locality [22]. This in turn helps support the households that created the brand [48]. Pienak et al. [49] determined that the product price did not significantly affect the consumers’ attitudes toward buying and consuming these products. However, their production value is significantly higher both in terms of their procurement and sales in the form different gastronomic products in catering facilities [50].

Regardless of the influence of global brands, the production, sale, and placement of TFPs in the hospitality and tourism industries has a significant economic benefit for the economy and for society. It provides positive benefits to producers and the local community as it ensures an additional income, has a favourable effect on diversification and different types of activities [34,47,51], and contributes to the decrease in population poverty [26,48].

#### 2.2.4. Touristic Factors

In the context of tourism development, TFPs preserve traditional food production, culture, identity, and heritage, highlighting the tourists’ interest in these products as significant factors of the local development of agriculture and handicraft production. They also contribute to the improvement and preservation of culinary culture and heritage [39,40]. Niedbala et al. [38] explain that the combination of TFPs and tourism as a part of local activities represents an investment in the future which affects the sustainability of gastronomy and economy [38]. Bassiere and Tibere [39] point out that tourists’ interest in such food is one of the key factors of local economic development which contributes to the development of food culture and heritage. It enables tourists to become familiar with less famous cultural heritage simply by means of ethnic restaurants’ offers.

A study carried out by Bryła [41] states that the characteristics that distinguish TFPs from conventional food include links to tradition, as well as sensory and health properties. Factors that can be singled out include natural taste, product quality, sales in the region of origin, and labelling. The most important factors in choosing these products are the traditional recipe, taste, and uniqueness of the product.

Tourists are much more interested in obtaining information about these products than the mass-produced products that can be found in the tourist offer. TFPs help create a general impression and form attitudes towards the tourist destination in question, which are aimed at economic development [22].

## 3. Methodology

### 3.1. The Research Location

This study was carried out in the Autonomous Province of Vojvodina, which represents an economically significant region in the northern part of the Republic of Serbia. This study was conducted on 300 respondents from the management sector of different catering facilities, because they are key figures in choosing and purchasing the ingredients necessary for the preparation of meals that are a part of the catering facilities’ offers. 

The selection of the facilities was based on random sampling. It is important to emphasize that the collection of data from employees in the hospitality industry was proportional to the total number of employees in the administrative areas of the region, in accordance with the data from the Statistical Office of the Republic of Serbia [52].

It is important to emphasize that the sample of respondents was part of more extensive research into TFPs, and that this paper presents results related to this issue.

Data collection was carried out from February to June 2022, with prior consent from the respondents for this type of survey, and with absolute anonymity. This means that, when collecting data, ethical principles were respected and the respondents voluntarily participated in the study.

### 3.2. Survey Questionnaire 

The set TFPct scale (TFPs in catering and tourism) was modelled after scales used in similar studies. The research statements were modelled on the research model set forth by Nguyen et al. [8] and included economic, sociocultural, and environmental factors. The statements were adapted to the studied area and to the characteristics of the TASQ TFPs.

In accordance with the topic of the study, statements from the field of food in tourism were added, as per Kalenjuk et al. [16,17]. Nineteen statements were included in the TFPct scale (shown in Table 4). The answers to the statements are defined on a Likert-type scale ranging from 1 to 5 (1—completely disagree to 5—completely agree). The statements include elements which refer to the decreased use of PVC packaging for TPFs, the impact of the reduction in transport and on pollution in the environment, the reduced use of preservatives, as well as the use of autochthonous plant types. In addition, the statements also include the impact of ecological awareness among employees and consumers, as well as the contribution these products make by being offered as part of the local cuisine, their impact on the economy of the region, and the promotion and marketing of local products in tourism. Based on the analysed studies, the statements aimed to determine the level of perception of the strengthening of social ties between the suppliers and buyers, provide an opportunity to the guests to familiarize themselves with the locality, the quality of the offer with a better rating for the hospitality facilities, and of course consider profitability. The aim of this study was to answer the following questions by means of different statistical analyses:

Q_1_: Will the proposed study determine the factors which are the subject of the study?

Q_2_: Is the set scale adequate for this type of TFP research in tourism?

Q_3_: Are there any changes needed in order to make the set scale more effective?

The main research question derived from these questions is:

Q_o_: Which elements from the set scale are important in TFP consumption, is there a factor that stands out significantly, and are there certain differences between the respondents?

### 3.3. Statistical Methods of Data Processing

The statistical analysis was performed in R version 4.1.2 using packages psych [53] for factor analysis and blorr [54] and rms [55] for the logistic regression [56]. The first part of the questionnaire included questions on the socio-demographic characteristics of the respondents, while a descriptive statistical analysis was used to statistically analyse these data.

The second part of the questionnaire included questions pertaining to the management sector of catering facilities, in particular their perception and selection of TFPs, in order to determine the factors which affect their views and decisions. It consists of 19 statements whose answers were valued on a Likert-type scale ranging from 1 to 5. A principal component analysis (PCA) was applied to determine the factors influencing the respondents’ decision to purchase TFPs for the needs of their catering facility.

The assumption underlying the factor analysis is the existence of a correlation between the input variables, i.e., that the correlation matrix should incorporate correlation coefficients that have a value above 0.3. Additionally, the strength of the correlation between the initial set of variables and the justification of the factor analysis application was tested using the Kaiser–Meier–Olkin (KMO) test and Bartlett’s test of specificity.

In order to apply a factor analysis, it is necessary to separate common factors from the correlation matrix, that is, to determine the number of factors that have common characteristics based on the percentage of variance. After separation, the non-rotated factor matrix is obtained, and then a rotation is performed, which more precisely defines the significance of each of the separated factors.

By rotating the factors, dimensionality is reduced and the optimal model containing a smaller number of factors is discovered. Factorial rotation was performed using the Varimax rotation with Keiser normalization.

Cronbach’s alpha was used to test the reliability of the study. The expected minimum value of Cronbach’s alpha which shows that the data are suitable for analysis is 0.6 [57].

After the separation of factors, the binary logistic regression method was used to determine which of the identified factors has the greatest impact on the decision to purchase TFPs.

The logistic regression model is based on cumulative logistic probability functions, while the dependent variable is dichotomous. If π represents the probability that something will not happen, then p/(1 − p) is the odds ratio. Apropos:

π/(1 − π) = e^(α + β1 × 1 + β2 × 2 + ⋯ βk × k)

If both sides of the above equation are logarithmic, with a natural logarithm, the following expression is obtained:

ln(π/(1 − π)) = α + β1 × 1 + β2 × 2 + ⋯βk × k

The obtained equation is called the logit and it is linear with the parameters ßi, i = 1…k. It can be seen that π belongs to the interval [0, 1], while the logit value ranges from (−∞, +∞), so it can be said that the logit function is the best choice for the representation of this function [58].

### 3.4. A Description of the Sample of Respondents

In order to comprehensively analyse the data, the starting point is the analysis of the socio-demographic characteristics of the respondents, the results of which are shown in Table 1. Based on the results of the descriptive statistical analysis, among the 300 respondents from the management sector, the percentage of male respondents was as high as 60%. When it comes to the age structure of the respondents, the largest percentage of respondents were 41 years old and older (36.7%), followed by respondents under the age of 30 (32%), and respondents aged 31 to 40 (31.3%).

According to the level of education, the highest percentage of respondents completed secondary school (70%). It is important to point out that slightly more than half of the respondents (50.7%) have an education in the field of catering and tourism. Based on their work experience in the hospitality industry, which is relevant for obtaining data from the respondents, the respondents were divided into three categories. The first category included those with up to 5 years of experience (27%), the second category included those with 6–10 years of experience (26%), the third category included those with 11–15 years of experience (17.7%), while the forth category included those with 16 and more years of experience (29.3%). Based on their work experience in their current facility of employment, the respondents were divided into three categories, which are fairly even in terms of members.

As previously mentioned, the focus was on obtaining data from the management sector of catering facilities. Among the respondents, most were deputy chefs (35%), followed by directors, i.e., owners of the facility (29%) and chefs (21%), while food and beverage managers were the least frequent (15%). Half of the respondents (50%) are employed in a la carte restaurants, 24.7% in fast food restaurants, 22.7% in other types of restaurants, and only 2.7% in mass catering restaurants.

## 4. Results

### 4.1. Factor Analysis

In the beginning, in order to determine whether the results are biased, Harman’s single-factor test was used [54]. The results of this test show that, when testing all the variables using a principal components analysis, the total extracted variance was less than 50% (42.75%), that is, there were no significant biased effects. Before the preparation of the factor analysis, the validity of the application was tested using the Kaiser–Meyer–Olkin test and Bartlett’s test of sphericity (Table 2).

The obtained value of the Kaiser–Meyer–Olkin coefficient was 0.93, which is higher than the minimum recommended value of 0.6 [52], and it can be considered adequate to use a factor analysis for the given set of variables. These results were confirmed by the results of Bartlett’s test of sphericity (*p* < 0.05), and it can be stated that there is a statistically significant correlation between the studied variables.

Moreover, it was noticed that the correlation matrix has a sufficient number of coefficients whose values are greater than 0.3, as well as a sufficient number of statistically significant correlation coefficients.

The principal components method was used to identify factors which are found in the correlation matrix. This method identifies groups of variables that have high correlation coefficients within the group and low correlation coefficient values with other groups. After the extraction of factors, the Varimax rotation was applied, and Table 3 shows the values for the four extracted factors.

Based on the values presented in this Table, it can be noticed that four factors with values higher than 1 were separated by the principle component method. These four factors explain 72.42% of the total variance.

Table 4 presents the descriptive statistics regarding the perception and choice of TFP by the management sector of catering facilities.

When taking into consideration environmental factors, the respondents agreed the most with the statement that using traditional products reduces the need to overuse PVC packaging and the least with the statement that meal preparation of traditional products raises ecological awareness among employees and consumers. In the case of socio-cultural factors, the respondents mostly agreed with the statements that offering meals made of traditional products gives guests an opportunity to get acquainted with the locality and that offering meals made of traditional products is a good marketing ploy. The economic factor they considered to be the most important is that offering meals made of traditional products increases meal demand, while the tourist factor that the respondents considered to be the most important is that traditional products can be a significant touristic attraction.

In the following part of the analysis, factor loading after rotation will be observed (Table 5). As a part of the named factors, factor saturation for each statement was observed, in order to confirm their role and significance. In presenting the results of the factor analysis, a finding was considered to be significant for a certain factor if the primary saturation was higher than 0.50.

Based on the results shown in Table 5, it can be seen that the first factor has the highest values of factor loading in the first five statements and was defined as the environmental factor. The second factor is defined through seven statements that refer to the impact that meal offers prepared from TFPs have on the social community and was labelled as the sociocultural one. 

The third factor is mostly defined by three statements that refer to the economic aspect of meals made of TFPs; therefore, this factor is labelled the economic factor. The last one is mostly defined by four statements that refer to the impact that meals prepared with TFPs have on the touristic offer, thus this factor is termed the touristic factor. 

Table 4 also shows that the statements’ factor loadings have different values for different factors. Based on their values, the ones that have the greatest impact on each factor could be separated. The statement which assumes that using TFPs reduces the use of PVC packaging (0.839) has the highest factor loading of the environmental factor, a reduction which is at the same time the greatest advantage of using TFPs. The second factor is most saturated by the statement that the meal offer of TFPs makes the local community proud of its products (0.764) and least saturated with the statement that the meal offer of TFPs represents a good marketing trick (0.580). The economic factor has the highest loading for the statement that refers to the meal offer of TFPs significantly influencing the sale values of meals (0.786). The touristic factor is most saturated with the statement that refers to TFPs leading to a higher quality tourist offer (0.851).

### 4.2. The Logistics Regression

The last part of the analysis includes testing which of the four factors affect the decision of the management sector of catering facilities to supply TFPs to their catering facilities. Therefore, a binary logistics regression model was used. The results of the Omnibus coefficient test showed that the model is well-adjusted with the data (χ^2^(1) = 5.580, *p* = 0.018). This result was confirmed by the Hosmer Lemeshow test (χ^2^(8) = 8.667, *p* = 0.371). The results of the regressive factor analysis that influenced the decision to purchase TFPs for catering facilities are shown in Table 6.

Based on the results of the logistic regression shown in Table 6, it can be observed that the economic factor (*p* < 0.05) is the only significant factor that influences the decision of the management sector of catering facilities to purchase TFPs. 

The only statistically significant characteristic that stood out when observing the socio-demographic characteristics of the respondents was the type of a facility (*p* < 0.05). The probability of the management sector in the hospitality industry purchasing TFPs for catering facilities differs significantly in relation to the type of facility. From the previous table it can be seen that the lowest probability (46.73%) for a TFP purchase is decided by the employees who work in mass catering restaurants, while the highest probability (76.65%) is that the TFPs will be purchased by employees of a la carte restaurants. 

## 5. Discussion

Based on the set TFPct scale of 19 items, we obtained answers to our set of auxiliary research questions (Q_1_, Q_2_, and Q_3_). They referred to our set scale being able to clearly define all four factors and as such not requiring the addition of any items or changes. The main research question (Q_O_) had the aim of determining which elements from the set scale have a significant role in the consumption of TFPs and whether there is a factor which stands out significantly. Additionally, it was meant to determine if there are certain differences among the respondents, which is presented and discussed in the ensuing text. 

The ecological benefits of TFP use are numerous. Just like other sectors of the economy, they are aimed at preserving the ecosystem and protecting the environment [26].

However, when analysing the perception of environmental factors with adequate statistical analyses, we obtained data that the respondents consider the reduction of PVC packaging to be the biggest advantage of TFP use, as contemporary industry uses plastic materials in mass production [31].

Observing the significance of socio-cultural factors in TFP use in the hospitality industry, when processing the data, it was noted that the management sector of the catering facilities believes, to a statistically significant extent, that the use of TFPs as food makes their community proud. This element specifically highlights the effects of TFPs as products of social trust, marked with a unique quality guarantee [59].

Marketing is an important step in the dissemination of information and the creation of an image of the characteristics of products found on the market [18,22]. The study showed that employees have the lowest perception of the significance of these products as a marketing element in business. Research has shown that the marketing opportunities of small- and medium-sized companies engaged in food production, which usually find it difficult to keep up their market presence next to large companies, are different and that they should be dealt with in more detail [18]. However, Bryla [41] emphasizes that making frequent references to tradition when marketing a locality is constantly growing.

Ghadban and Fayad [60] affirm that the structure of the offer on a menu represents a primary determinant of profitability in catering facilities. The data obtained regarding economic factors showed that caterers claim meals made of TFPs significantly influence their sales values, which is justifiable due to the nature of the production process [50]. When processing the data, it was determined that only the economic factor has a significant impact on the decision of the management sector of catering facilities to purchase TFPs. These data included questions about marketing values, increase in demand, and profitability that can be gained in offering meals made of TFPs. Marketing these products provides extra income to a community and, as stated by NaSonkhla and Somboonsuke [51], it has a favourable effect on diversification and different types of activities. Based on the data obtained, it can be seen that the employees in catering facilities are aware that the offer and sale of TFPs significantly contributes to a reduction in population poverty, helps promote sustainable agriculture and handicraft production, reduces regional economic gaps, and increases the local economy, which was confirmed in the studies of other authors [23,44,45,48].

The study of touristic factors showed that employees in the hospitality industry perceive the TFP offer in catering facilities as products that influence the quality of the touristic offer. The quality of the touristic offer relates to the authenticity of the gastronomic offer of a locality as extremely important for the unique gastro-touristic experience [61] inspired by TFPs.

The study showed that the probability that a management-sector employee will decide to buy TFPs for his/her catering facility needs significantly depends on the type of facility. The lowest probability of deciding to purchase TFPs was found for employees who work in mass catering restaurants, such as workers’ restaurants, canteens, school restaurants, hotel complexes, care centres, hospitals, and other similar facilities. The highest probability is that TFPs will be purchased by employees who work in a la carte restaurants, which have traditional meals on their menus. These are facilities whose business is aimed at consumers who seek a unique gastronomic experience, which includes tourists as well. The data obtained show that employees of catering facilities are aware that the financial performance of a restaurant is stipulated by its offer of authenticity [41,42], which is associated with TFPs. 

**Figure 1:**
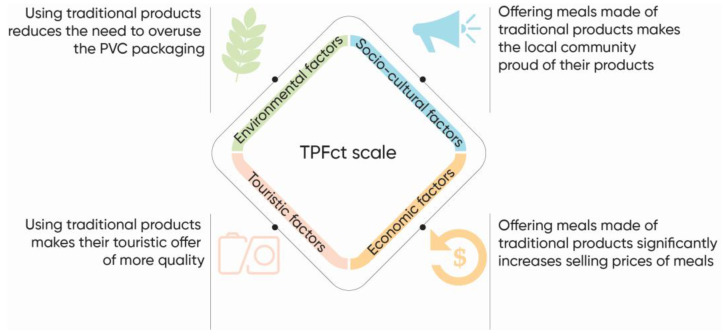
Conceptualization of the study design (Authors: Đerčan, 2023).

Figure 1 provides a clear presentation of the defined factors and results obtained.

## 6. Conclusions

The analyses carried out on the set TFPct scale proved to be adequate for this type of research, which clearly distinguished all four factors and gave concrete results for each of them.

Based on the results, it was concluded that the use of TFPs significantly reduces the use of PVC packaging. Catering facility employees claim that the use of PVC packaging in procurement, supply, and consumption is significantly lower in TFPs than in mass-produced products. This has emerged as the most prominent environmental factor.

The impact the food marketed in tourism has on the awareness of both consumers and producers is very important. The placement and popularization of TFPs in tourism is increasing because they are products that the community is proud of, and according to the respondents, this is the most important socio-cultural factor. In other words, the respondents consider that the use of TFPs will influence consumer awareness by increasing their gastronomic recognition and the frequency of use of TFPs in hospitality and the touristic offer. The use of TFPs in catering facilities specifically increases the quality of the offer in tourism. By implementing products that are traditional and unique for a certain area, the offer of the catering facility is improved and diversified, which significantly affects the quality of the offer itself.

Similarly, the use of TFPs in certain meal preparations significantly influences the price of the meal. These products lead the consumers to perceive them as more expensive due to their characteristic preparation and authenticity; therefore, their higher price in relation to other meals is justified. 

Looking at the influence of all the factors from the TFPct scale, it can be concluded that economic factors related to the increase in sales value, demand, and profitability have the greatest influence on the use and consumption of TASQ TFPs. In addition to having a significant impact on the economy of the catering facility itself (primarily a la carte restaurants, which have also expressed great interest), the use of TFPs in the preparation of meals also has an impact on the economy of the entire community and population, spreading the influence of the hospitality industry to all other branches of the economy.

### 6.1. Practical Implications

Basically, the study updates the theoretical facts in the field of TFPs. The study, based on the set TFPct scale, indicates the possibility of implementing the scale in different agricultural-gastro-touristic localities, in order to obtain data that will help in the placement and utilization of TFPs in the hospitality and tourism industries.

The results obtained provide useful information to public and private institutions in the food sector, hospitality industry, and tourism industry, as well as to creators of legal regulations and strategies in the field. The data can help improve TFP product placement on the catering-touristic market and therefore support the offer in the entire supply and demand chain. Based on the results which pertain to the analysed factors, clear guidelines can be observed for the better placement and utilization of TFPs, which would altogether have a more significant economic, ecological, social, and touristic utilization.

### 6.2. Research Limitations

Catering facilities which provide food and beverages spend considerable amounts of food and agricultural products. The limitations of this study refer to the possibility of collecting more detailed data on the types and quantities of TFPs that catering facilities purchase and offer their guests. Obtaining such data would require a separate study which would also require insight into internal business documentation. These types of data would provide valuable information for further activities in the business sector. 

### 6.3. Indications for Future Research

An indication for future research would be to conduct qualitative research among employees in the hospitality industry. Qualitative studies of the management sectors of catering facilities would provide insight into individual segments and provide much more detailed data, such as the types and quantities of products they purchase, as well as precise data on the types of products and the reasons for their low consumption and selection.

Similar research on TFPs could be conducted among guests of restaurants and tourists, providing more tangible and concrete data on the importance of the TFP offer in the hospitality industry and tourist industry of each studied locality. Research could focus on the management of gastronomic satisfaction as an essential element in the development of tourism and in improving satisfaction [62].

## Figures and Tables

**Table 1:** The socio-demographic characteristics of the respondents.

Variables	Categories	n	Percentage
Gender	male	180	60.0
	female	120	40.0
Age	Up to age 30	96	32.0
	Ages 31–40	94	31.3
	41 years old and older	110	36.7
Education level	Secondary	210	70.0
	Community college associate degree	47	15.7
	University bachelor’s degree	41	13.7
	Master/PhD	2	0.7
Field of education	Hospitality, tourism	152	50.7
	Economy, law, management	65	21.7
	Food technology, agriculture, chemistry	25	8.3
	Other fields	58	19.3
Work experience	0–5 years	81	27.0
	6–10 years	78	26.0
	11–15 years	53	17.7
	16 years and more	88	29.3
Work experience in the facility	0–2 years	110	36.7
	3–5 years	86	28.7
	6 years and more	104	34.7
Working position	Chef	63	21.0
	Deputy chef	105	35.0
	Food and beverages manager	45	15.0
	Owner of the facility	87	29.0
Facility type	Mass catering restaurants	8	2.7
	A la carte restaurant	150	50.0
	Fast food restaurant	74	24.7
	Other types of restaurants	68	22.7

Source: Edited by the author.

**Table 2:** The Kaiser–Meyer–Olkin (KMO) test and Bartlett’s test of justification of the factor analysis.

Kaiser–Meyer–Olkin Measure of Sampling Adequacy.		0.930
Bartlett’s Test of Sphericity	Approx. Chi-Square	3600.729
	df	171
	Sig.	0.000

Source: Edited by the author.

**Table 3:** The total variance explained.

Components	Initial Eigenvalues			Extraction Sums of Squared Loading			Rotation Sums of Squared Loading		
	Total	% of Variance	Cumulative %	Total	% of Variance	Cumulative %	Total	% of Variance	Cumulative %
1	9.493	49.965	49.965	9.493	49.965	49.965	4.162	21.905	21.905
2	1.839	9.680	59.645	1.839	9.680	59.645	3.912	20.589	42.494
3	1.111	5.849	72.418	1.111	5.849	72.418	2.431	12.795	72.418
4	1.316	6.924	66.568	1.316	6.924	66.568	3.255	17.129	59.623

Source: Edited by the author.

**Table 4:** Descriptive statistics of the variables used in the factor analysis.

Variables	Mean	Standard Deviation
Using traditional products reduces the need to overuse PVC packaging	4.187	1.031
Using traditional products reduces the negative impact of traffic on environmental pollution	4.083	1.074
Using traditional products affects preservation of the environment due to reduced use of preservatives	4.078	1.043
Using traditional products improves the use of domestic raw materials	4.103	1.001
Meal preparation of traditional products raises ecological awareness in employees and consumers	3.920	1.076
Offering meals made of traditional products makes the local community proud of their products	4.170	0.965
Offering meals made of traditional products supports the region’s economy	4.210	0.869
Offering meals made of traditional products affects the promotion of local products	4.353	0.912
Offering meals made of traditional products uses community resources completely	4.001	0.954
Offering meals made of traditional products strengthens social links between suppliers and consumers	4.100	0.983
Offering meals made of traditional products gives an opportunity to guests to become acquainted with the locality	4.387	0.883
Offering meals made of traditional products is a good marketing trick	4.387	0.872
Offering meals made of traditional products significantly increases the selling prices of meals	4.107	0.924
Offering meals made of traditional products increases meal demand	4.163	0.931
Offering meals made of traditional products is profitable	3.800	0.925
Traditional products can represent a significant touristic attraction	4.490	0.772
Using traditional products makes their touristic offer one of higher quality	4.390	0.787
Offering meals made of traditional products gives a catering facility higher ratings	4.320	0.845
Using traditional products promotes and represents a local tradition in tourism	4.370	0.854

Source: Edited by the author.

**Table 5:** Factor loading after rotation.

Variables	Component			
	1	2	3	4
Environmental factors				
Using traditional products reduces the need to overuse PVC packaging	0.839			
Using traditional products reduces the negative impact of traffic on environmental pollution	0.836			
Using traditional products affects preservation of the environment due to reduced use of preservatives	0.825			
Using traditional products improves the use of domestic raw materials	0.750	0.311		
Meal preparation of traditional products raises ecological awareness in employees and consumers	0.709	0.329		
Socio-cultural factors				
Offering meals made of traditional products makes the local community proud of their products		0.764		
Offering meals made of traditional products supports the region’s economy		0.709	0.394	
Offering meals made of traditional products affects the promotion of local products	0.344	0.691		0.397
Offering meals made of traditional products uses community resources completely	0.411	0.660		
Offering meals made of traditional products strengthens social links between suppliers and consumers	0.541	0.624		
Offering meals made of traditional products gives an opportunity to guests to become acquainted with the locality		0.600		0.472
Offering meals made of traditional products is a good marketing trick		0.580	0.310	0.410
Economic factors				
Offering meals made of traditional products significantly increases the selling prices of meals			0.786	
Offering meals made of traditional products increases meal demand			0.775	
Offering meals made of traditional products is profitable		0.349	0.691	
Touristic factors				
Traditional products can represent a significant touristic attraction				0.851
Using traditional products makes their touristic offer one of higher quality				0.756
Offering meals made of traditional products gives a catering facility higher ratings			0.382	0.711
Using traditional products promotes and represents a local tradition in tourism		0.470		0.670
Cronbach’s Alpha	0.912	0.910	0.869	0.780

Source: Edited by the author.

**Table 6:** The logit model results.

Variables	B	S.E.	Wald	df	Sig.	Exp(B)	Probability
Environmental factors	−0.126	0.164	0.595	1	0.441	0.881	46.84%
Socio-cultural factors	0.153	0.163	0.879	1	0.348	1.165	53.81%
Economic factors	−0.365	0.156	5.486	1	0.019	0.695	41.00%
Touristic factors	−0.180	0.144	1.555	1	0.212	0.835	45.50%
Gender–male	−0.146	0.338	0.188	1	0.665	0.864	46.35%
Age	−0.022	0.024	0.831	1	0.362	0.978	49.44%
Level of education			0.676	3	0.879		
Secondary	17.825	28,419.885	0.000	1	0.999	4.129	80.50%
Community college associate degree	18.146	28,419.885	0.000	1	0.999	2.361	70.24%
University bachelor’s degree	18.076	28,419.885	0.000	1	0.999	9.394	90.85%
Work experience in the hospitality industry	−0.039	0.034	1.294	1	0.255	0.962	49.03%
Years of service in a catering facility	−0.010	0.044	0.052	1	0.819	0.990	49.75%
Position			6.803	3	0.078		
Chef	1.121	0.501	5.008	1	0.025	3.067	75.41%
Deputy chef	0.500	0.474	1.111	1	0.292	1.649	62.25%
Food and beverages manager	1.076	0.513	4.404	1	0.036	2.933	74.57%
Type of facility			10.883	3	0.012		
Mass catering restaurant	−0.131	0.948	0.019	1	0.890	0.877	46.73%
A la carte restaurant	1.134	0.973	1.359	1	0.244	3.108	75.65%
Fast food restaurant	0.680	0.976	0.486	1	0.486	1.975	66.39%
Constant	−19.030	28,419.885	0.000	1	0.999	0.000	

Source: Edited by the author.

## Data Availability

Data are available upon request due to restrictions.
